# Determination of Peripheral Neuropathy Prevalence and Associated Factors in Chinese Subjects with Diabetes and Pre-Diabetes – ShangHai Diabetic neuRopathy Epidemiology and Molecular Genetics Study (SH-DREAMS)

**DOI:** 10.1371/journal.pone.0061053

**Published:** 2013-04-16

**Authors:** Bin Lu, Ji Hu, Jian Wen, Zhaoyun Zhang, Linuo Zhou, Yiming Li, Renming Hu

**Affiliations:** 1 Department of Endocrinology, Huashan Hospital, Fudan University, Shanghai, China; 2 Department of Endocrinology, The Second Affiliated Hospital of Soochow University, Jiangsu, China; 3 Department of Endocrinology, Jing’An District Centre Hospital of Shanghai, Shanghai, China; German Diabetes Center, Leibniz Center for Diabetes Research at Heinrich Heine University Duesseldorf, Germany

## Abstract

**Objective:**

This study determined the prevalence and factors associated with peripheral neuropathy (PN) in subjects with diabetes mellitus, impaired glucose regulation (IGR), and normal glucose tolerance (NGT) in a community-based Chinese population.

**Research Design and Methods:**

A total of 2035 subjects in Shanghai were classified as having NGT, IGR, or diabetes. All subjects underwent complete foot examination. PN was assessed according to the neuropathy symptom and neuropathy disability scores. Binary logistic regression was performed to analyze the contributions of factors to PN.

**Results:**

The prevalence of PN was 8.4%, 2.8%, and 1.5% in diabetes mellitus, IGR, and NGT subjects, respectively (*P*<0.05 for diabetes vs. NGT, and IGR). The subjects with known diabetes had the highest frequency of PN (13.1%). Among the subjects without diabetes, those with PN were older, had a higher waist circumference and 2-h postprandial plasma glucose levels, and were more likely to be hypertensive. Among the IGR subjects, other than age, the 2-h postprandial plasma glucose level was an independent factor significantly associated with PN. Meanwhile, among the subjects with diabetes, PN was associated with fasting plasma glucose, duration of diabetes, and decreased estimated glomerular filtration rate.

**Conclusions:**

The prevalence of PN is slightly higher in individuals with IGR than that in individuals with NGT, but small fibre damage in IGR as the earliest nerve fibre deficit may be underestimated in our study. As an independent risk factor, postprandial plasma glucose level may be an important target for strategies to prevent or improve PN in IGR subjects.

## Introduction

Diabetes is a leading public health problem in China and imposes heavy economic burdens on Chinese patients [1]. An estimated 92 million people in China have type 2 diabetes, which is the largest number worldwide.

Microvascular and macrovascular complications are the leading causes of diabetes-associated morbidity and mortality. Our previous study revealed that up to 61.8% of patients diagnosed with type 2 diabetes in downtown Shanghai have peripheral neuropathy (PN) according to the evaluation of vibration perception threshold and 10-g Semmes–Weinstein monofilament [2]. However, there are limited data concerning the prevalence of neuropathies in the pre-diabetes population, especially in Han Chinese.

In China, a study on the She ethnic minority group revealed the prevalence of polyneuropathy in impaired fasting glucose (IFG), impaired glucose tolerance (IGT), IFG/IGT, and diabetes mellitus (DM) to be 16.1%, 13.1%, 18.6%, and 28.4% respectively, which are greater than that in patients with normal glucose tolerance (NGT) [3]. A study on a Western population revealed that the prevalence of neuropathic pain was 13.3%, 8.7%, 4.2%, and 1.2% in normal subjects and subjects with diabetes, IGT, and IFG, respectively [4]. In the same cohort, the overall neuropathy risk was evaluated using the Michigan Neuropathy Screening Instrument; neuropathy was present in 28%, 13%, 11.3%, and 7.4% of diabetes, IGT, IFG, and control subjects, respectively [5]. All of the studies mentioned above indicate that the prevalence of polyneuropathy is slightly higher in individuals with IGT and IFG than that in individuals with NGT. However, some studies indicate that IGT may be not associated with peripheral nerve dysfunction [6].

Several recent studies report an association between IGT and PN [7], [8]. The observation of a high prevalence of IGT in idiopathic neuropathy is nearly uniform. Of the patients with clinically confirmed, cryptogenic, predominantly sensory neuropathy, 56% have abnormal oral glucose tolerance test (OGTT) results, with 36% and 20% having IGT and diabetes, respectively. These percentages are 2- to 3-fold greater than that reported in the National Health and Nutrition Examination Survey (NHANES) III study, which reports an IGT prevalence of 15.8% among 2844 participants aged 40–74 years. Small studies evaluating nerve function describe abnormal nerve conduction study results [9], sympathetic skin responses [10], and laser Doppler flare [11]. Although IGT-associated neuropathy has been confirmed, it may be milder than that associated with diabetes [12], [13].

Significant differences in risk factors associated with the onset or progression of neuropathy may exist among various populations. Despite this, few studies have been performed to distinguish the risk factors for PN among various glucose metabolisms in Asian races, especially in the Han Chinese population.

Therefore, the present study assessed the prevalence of PN and its associated differential risk factors in individuals with diabetes and pre-diabetes in a Han Chinese cohort.

## Research Design and Methods

### Study Population

One of the purposes of the Shanghai diabetic neuropathy epidemiology and molecular genetics study (SH-DREAMS) was to evaluate PN in Han Chinese subjects with diabetes and pre-diabetes. All nonpregnant community members aged >25 years without type 1 diabetes or renal failure were invited to participate, and a total of 2149 voluntary individuals aged >25 years were enrolled from 2 communities: Gongkang and Sitang. Diabetes and pre-diabetes were diagnosed according to the OGTT. Among the analyzed population (n = 2035), 534 and 1043 subjects were diagnosed with diabetes and impaired glucose regulation (IGR), respectively. All individuals were recruited in the SH-DREAMS from July 2011 to May 2012. Written informed consent was obtained from all participants. All protocols were approved by Huashan Hospital ethics committee.

### Anthropometric Measurements

All participants were asked to fill out a questionnaire to collect their demographic information as well as medical history of diabetes and related diseases. Physical examination included measurement of height, weight, waist circumference, hip circumference, and blood pressure. Blood pressure was measured thrice using a standard mercury sphygmomanometer and then averaged. Hypertension was defined as systolic blood pressure ≥140 mmHg and/or diastolic blood pressure ≥90 mmHg (WHO 1999) [14] or as current use of blood pressure-lowering medication. BMI was calculated as weight divided by height squared (kg/m^2^). Obesity was defined as BMI ≥30 kg/m^2^ (WHO 1997) [15]. The waist-to-hip ratio was calculated as the ratio of the waist circumference to the hip circumference. Other information such as living habits was also collected.

### Laboratory Measurements

After a fasting venous blood sample was collected, each participant received a 75-g OGTT, except for those with a validated history of DM with 100 g steamed bread meal test. 100 g steamed bread meal test, though not a standard test in diabetes research or care, can avoid severe glucose fluctuation in those with a validated history of DM. Plasma glucose levels were measured using the glucose oxidase method. Levels of glycated hemoglobin (HbA1C) were estimated by high-pressure liquid chromatography using an analyzer (HLC-723G7, Tosoh Corporation, Japan). Levels of serum urea nitrogen, creatinine, and uric acid as well as lipid profiles including measurements of total cholesterol, triacylglycerol, HDL-cholesterol, and LDL-cholesterol were measured on a Hitachi 7600 analyzer using an enzymatic assay. Urinary albumin was measured by immunonephelometry. The urinary creatinine concentration was measured on a Hitachi 7600 analyzer using an enzymatic assay.

### Definitions

Diabetes and IGR were diagnosed according to the standards set by the American Diabetes Association (ADA) in 2012 [16]. IGR is now designated as “increased risk for diabetes,” and it can be defined as fasting plasma glucose (5.6–6.9 mmol/L), 2-h postprandial glucose level in the 75-g OGTT (7.8–11.0 mmol/L), and/or HbA1C level (5.7%–6.4%).

### PN Screening and Assessment

Neuropathy Deficit Score (NDS): Neuropathic deficits in the feet were determined using the NDS, derived from the examination of vibration (using a 128-Hz tuning fork), pin-prick sensation (using Neurotip), temperature sensation (using warm and cool rods), and Achilles tendon reflex (using a tendon hammer). The 3 perceptions were scored 0 if present and normal, and 1 if absent, reduced, or uncertain. On either side, the ankle reflex was scored 0 if present and normal, and 2 if absent [17]. The maximum score was 10. The severity of neuropathy disability was graded as follows: mild (scores: 3–5), moderate (scores: 6–8), and severe (scores: 9–10).

Neuropathy Symptom Score (NSS): All patients were asked whether they experienced pain or discomfort in their legs. A description of burning, numbness, or tingling was assigned a score of 2, and fatigue, cramping, or aching was assigned a score of 1. If the patient described the symptoms as occurring in their feet, calves, and elsewhere, scores of 2, 1, and 0 were assigned, respectively. Nocturnal exacerbation of symptoms was scored as 2; exacerbation of symptoms during the day as well as night was scored as 1, and exacerbation of symptoms during the daytime alone was scored as 0. If the symptoms had ever woken the patient from sleep, a score of 1 was assigned. The patients were asked if any maneuver could reduce their symptoms; walking was assigned a score of 2, standing 1, and sitting or lying down 0. Thus, the maximum symptom score was 9, and the severity of symptoms was graded as follows: mild (scores: 3–4), moderate (scores: 5–6), and severe (scores: 7–9) [18].

The diagnosis of PN depends on both subjective symptoms and signs of neuropathy. We defined PN as at least moderate signs with or without symptoms (NDS ≥6), or mild signs with moderate symptoms (NDS ≥3 and NSS ≥5) [17], [19].

### Statistical Analysis

All statistical analyses were performed using SPSS software version 16.0. Normally distributed and continuous variables are expressed as mean ± standard deviation, and non-normally distributed variables are presented as medians (25% and 75% quartiles). To assess the differences among NGT, IGR, and diabetes with respect to quantitative data, 1-way ANOVA with SNK analysis was used. The Kruskal–Wallis H or χ^2^ test was used for comparisons involving non-normally distributed data. Binary logistic regression was performed to analyze the contributions of the risk factors to the presence of PN. Odds ratios (ORs) and their 95% confidence intervals (CIs) were calculated based on the logistic regression models and used to examine the association between PN and associated factors. The OR corresponds to a 1 unit change in categorical factors associated with PN. A positive or negative association is indicated if the OR is significantly (p<0.05) larger or smaller than 1, respectively. A 2-tailed *P-*value less than 0.05 was considered significant.

## Results

### Subject Characteristics

There were a total of 2035 subjects, including 728 men and 1307 women, with an average age of 61.5±10.1 years. These subjects were classified on the basis of the results from the 75-g OGTT according to the recent ADA criterion as follows: NGT, n = 458; IGR, n = 1043; and diabetes, n = 534. A total of 26.2% of subjects had type 2 diabetes, and 54.5% of them had known diabetes before they were tested. The subjects with newly diagnosed diabetes were younger than those with known diabetes (62.9±9.4 vs. 65.0±10.2 years) and had higher frequencies of microalbuminuria (31.4% vs. 23.0%) and macroalbuminuria (6.7% vs. 1.3%) and lower HbA1C levels (median, 6.5% vs. 7.1%).

The percentages of current smokers were similar among subjects with NGT (14.8%), IGR (13.0%), and diabetes (15.4%) (Table 1). Age, BMI, waist circumference, waist-to-hip ratio, and levels of HbA1C, total cholesterol, triglycerides, serum creatinine, and uric acid were significantly higher in the diabetes and IGR subjects than that in the NGT subjects. Systolic blood pressure, diastolic blood pressure, and LDL-cholesterol were significantly higher among the diabetes subjects than that in the NGT subjects. Albuminuria and hypertension were much more prevalent in diabetes subjects than that in NGT subjects. HDL-cholesterol and the evaluated glomerular filtration rate (eGFR) were significantly lower in the diabetes and IGR subjects than that in the NGT subjects.

**Table 1:** Clinical characteristics of study population by group of normal glucose tolerance (NGT),impaired glucose regulation(IGR) and diabetes.

Parameters	NGT(N = 458)	IGR(N = 1043)	DM(N = 534)	Total(N = 2035)
Sex(men/women)	141/317	357/686	230/304† ‡	728/1307
Age(years)	59.7±11.2	61.0±9.4†	64.0±9.9† ‡	61.5±10.1
Current smoker	68(14.8%)	136(13.0%)	82(15.4%)	286(14.1%)
BMI(kg/m2)	23.5±3.1	24.3±3.4†	25.5±3.8† ‡	24.4±3.5
Waist circumference(cm)	82.5±9.2	85.5±9.7†	90.4±10.2† ‡	86.1±10.1
Waist-to-hip ratio	0.87±0.06	0.88±0.07†	0.91±0.08† ‡	0.89±0.07
Blood pressure(mmHg)				
Systolic	124.3±18.5	126.3±18.8	135.7±21.1† ‡	128.3±19.8
Diastolic	78.7±10.4	79.8±10.3	81.6±10.7† ‡	80.0±10.5
FPG(mmol/L)	4.47±0.55	5.41±0.81†	7.40±2.78† ‡	5.72±1.89
2hPG(mmol/L)	5.70±1.15	7.23±1.71†	12.43±4.33† ‡	8.24±3.65
HbA_1C_ (%)	5.36±0.21	5.76±0.33†	7.14±1.50† ‡	6.03±1.06
HOMA-IR	1.04(0.76–1.46)	1.38(0.99–1.95)†	2.10(1.41–3.29)† ‡	1.42(0.97–2.10)
Total cholesterol (mmol/L)	5.22±1.00	5.41±0.97†	5.48±1.23†	5.38±1.06
Triglyceride (mmol/L)	1.46±0.93	1.67±0.95†	2.05±1.57† ‡	1.72±1.16
HDL-cholesterol (mmol/L)	1.40±0.33	1.36±0.31†	1.26±0.28† ‡	1.34±0.31
LDL-cholesterol (mmol/L)	3.06±0.73	3.12±0.69	3.23±0.85† ‡	3.14±0.75
Creatinine (µmol/L)	79.93±14.74	83.41±17.12†	86.98±25.53† ‡	83.56±19.38
eGFR (MDRD equation)	76.97±12.98	73.71±12.23†	72.88±13.94†	74.22±12.95
Uric acid(µmol/L)	289.58±75.86	301.90±79.06†	314.47±82.38† ‡	302.43±79.68
Urine ACR				
Microalbuminuria(%)	92(20.6%)	166(16.8%)	143(27.6%)† ‡	401(20.5%)
Macroalbuminuria(%)	6(1.3%)	14(1.4%)	22(4.2%)† ‡	42(2.1%)
Hypertension	194(42.4%)	469(45.0%)	359(67.2%)† ‡	1022(50.2%)
Obesity	13(2.8%)	61(5.8%)†	60(11.2%)† ‡	134(6.6%)
Medication				
Antihypertensive	98(22.3%)	231(23.0%)	219(43.3%)† ‡	548(28.1%)
ACEI/ARB	27(6.1%)	72(7.2%)	74(14.6%)† ‡	173(8.9%)
Lipid lowering	14(3.1%)	11(1.1%)†	14(2.6%)	39(1.9%)

Categorical variables were expressed as numbers.

Continuous variables were expressed as mean±SD.

NGT, normal glucose tolerance; IGR, impaired glucose regulation; DM, diabetes mellitus; BMI, body mass index; FPG, fasting plasma glucose; 2hPG,2-h postprandial plasma glucose; HbA1c, glycated hemoglobin A1c; HDL, high density lipoprotein; LDL, low-density lipoprotein; eGFR, estimated glomerular filtration rate; ACR, albumin-to-creatinine ratio; ANOVA, analysis of variance; SNK, Student Newman–Keuls; ACEI, angiotensin converting enzyme inhibitor; ARB, angiotensin receptor blocker.

† P<0.05 diabetes or IGR versus NGT, based on one-way ANOVA with SNK analysis, Kruskal–Wallis H test or χ^2^ test, as appropriate.

‡ P<0.05 diabetes versus IGR based on one-way ANOVA with SNK analysis, Kruskal–Wallis H test or χ^2^ test, as appropriate.

In diabetic patients, compared to subjects without PN (Table 2), subjects with PN were older, had higher HbA1C levels, and had macroalbuminuria more often (18.2% vs. 3.0%, *P*<0.05). Among the subjects without diabetes, those with PN were older, had a higher waist circumference and 2-h postprandial plasma glucose levels, and were more likely to be hypertensive, especially with respect to systolic blood pressure (133.1±22.7 vs. 125.5±18.6 mmHg, *P*<0.05).

**Table 2:** Basic features of subjects with and without peripheral neuropathy.

	Non-diabetes		Diabetes	
	Non-PN (n = 1465)	PN (n = 36)	Non-PN (n = 489)	PN (n = 45)
Sex(men/women)	489/976	9/27	217/272	13/32
Age(years)	60.3±9.9	72.4±9.8†	63.5±9.8	69.6±9.5†
Current smoker	202(13.8%)	2(5.6%)	78(16.0%)	4(8.9%)
BMI(kg/m2)	24.0±3.3	24.5±3.9	25.5±3.8	25.8±4.3
Waist circumference(cm)	84.5±9.6	88.4±11.9†	90.2±10.2	92.5±10.5
Waist-to-hip ratio	0.88±0.06	0.92±0.15†	0.91±0.08	0.92±0.05
Blood pressure(mmHg)				
Systolic	125.5±18.6	133.1±22.7†	135.3±20.9	139.4±23.0
Diastolic	79.5±10.3	78.0±11.5	81.7±10.7	79.7±11.0
FPG(mmol/L)	5.12±0.86	5.11±0.85	7.34±2.67	8.09±3.77†
2hPG(mmol/L)	6.72±1.70	8.06±1.71†	12.34±4.29	12.34±4.66
HbA_1C_ (%)	5.64±0.35	5.68±0.39	7.10±1.49	7.49±1.59†
HOMA-IR	1.28(0.90–1.81)	1.36(0.87–1.74)	2.10(1.35–3.27)	2.09(1.63–3.76)†
Total cholesterol (mmol/L)	5.36±0.99	5.13±0.84	5.48±1.22	5.46±1.34
Triglyceride (mmol/L)	1.61±0.95	1.41±0.69	2.06±1.57	1.91±1.60
HDL-cholesterol (mmol/L)	1.36±0.31	1.32±0.34	1.26±0.28	1.23±0.30
LDL-cholesterol (mmol/L)	3.10±0.71	3.04±0.58	3.22±0.84	3.30±0.90
Creatinine (µmol/L)	82.31±16.53	84.08±15.33	85.45±18.41	103.53±61.87†
eGFR (MDRD equation)	74.85±12.55	68.46±11.22†	73.80±13.20	62.79±17.56†
Uric acid(µmol/L)	298.18±78.41	296.64±73.52	313.86±82.49	321.11±81.82
Urine ACR				
Microalbuminuria(%)	245(17.5%)	13(37.1%)†	133(28.1%)	10(22.7%)
Macroalbuminuria(%)	18(1.3%)	2(5.7%)†	14(3.0%)	8(18.2%)†
Hypertension	639(43.6%)	24(66.7%)†	328(67.1%)	31(68.9%)
Obesity	70(4.8%)	4(11.1%)	57(11.7%)	3(6.7%)
Medication				
Antihypertensive	317(22.4%)	12(38.7%)†	199(43.1%)	20(45.5%)
ACEI/ARB	98(6.9%)	1(3.2%)	68(14.7%)	6(13.6%)
Lipid lowering	24(1.6%)	1(2.8%)	11(2.2%)	3(6.7%)

Continuous variables were expressed as mean±SD or median (25% and 75% quartile) for non-normally distributed variables.

PN, peripheral neuropathy; BMI, body mass index; FPG, fasting plasma glucose; 2hPG,2-h postprandial plasma glucose; HbA1c, glycated hemoglobin A1c; HDL, high density lipoprotein; LDL, low-density lipoprotein; eGFR, estimated glomerular filtration rate; ACR, albumin-to-creatinine ratio; ACEI, angiotensin converting enzyme inhibitor; ARB, angiotensin receptor blocker.

† P<0.05 No PN versus PN, based on Independent-Samples T-test, Kruskal– Wallis H-test, or χ2 test, as appropriate.

### PN Prevalence

As shown in Table 3, 4.0% of the 2035 participants were diagnosed with PN. The prevalence of PN in diabetes, IGR, and NGT subjects was 8.4%, 2.8%, and 1.5%, respectively; the prevalence of PN in subjects with diabetes was significantly greater than that in IGR and NGT subjects, although there was no significant difference between IGR and NGT subjects (χ^2^∶ 2.131, *P* = 0.199). The subjects with known diabetes had the highest frequency of PN (13.1%).

**Table 3:** The prevalence of PN in diabetes and impaired glucose regulation.

		DM			
	Total(n = 2035)	Known(n = 291)	Newly-diagnosed(n = 243)	IGR(n = 1043)	NGT(n = 458)
PN	81(4.0%)	38(13.1%)	7(2.9%)	29(2.8%)	7(1.5%)
NDS					
NDS mild	144(7.1%)	34(11.7%)	18(7.4%)	67(6.4%)	25(5.5%)
NDS moderate	50(2.5%)	25(8.6%)	3(1.2%)	18(1.7%)	4(0.9%)
NDS severe	3(0.1%)	3(1.0%)	0(0.0%)	0(0.0%)	0(0.0%)
NSS					
NSS mild	508(25.0%)	75(25.8%)	57(23.5%)	258(24.7%)	118(25.8%)
NSS moderate	368(18.1%)	78(26.8%)	39(16.0%)	176(16.9%)	75(16.4%)
NSS severe	41(2.0%)	10(3.4%)	3(1.2%)	21(2.0%)	7(1.5%)

Data are expressed as number (%). For categories with an absolute count of subjects less than five, prevalence or percentage was not provided.

IGR, impaired glucose regulation; NGT, normal glucose tolerance; NDS, Neuropathy Deficit Score; NSS, Neuropathy Symptom Score.

NDS was graded based on scores as follows: mild (3–5), moderate (6–8), and severe (9–10).

NSS was graded based on scores as follows: mild (3–4), moderate (5–6), and severe (7–9).

**Table 4:** Multiple logistic regression analysis using PN as a dependent variable among the NGT, IGR and DM subjects.

	Total (n = 2035)		IGR(n = 1043)		Known DM(n = 291)	
	OR (95% CI)	P	OR (95% CI)	P	OR (95% CI)	P
Age(years)	1.088 (1.055–1.122)	<0.001	1.096(1.033–1.163)	0.002*	1.039(0.991–1.089)	0.110
Gender (Female)	1.697(0.606–4.637)	0.369	1.497(0.508–4.417)	0.464	2.047(0.851–4.923)	0.110
Current Smoker (Yes)	0.532(0.203–1.391)	0.198	0.758(0.140–4.118)	0.995	0.382(0.094–1.559)	0.180
Waist Circumference (cm)	1.024(1.000–1.049)	0.054	1.014(0.968–1.063)	0.551	1.027(0.991–1.065)	0.145
Hypertension (Yes)	1.357 (0. 711–2.589)	0.355	1.867(0.625–5.577)	0.263	0.932(0.349–2.491)	0.888
Systolic BP (mmHg)	0.998(0.982–1.014)	0.786	0.995(0.967–1.024)	0.732	0.997(0.974–1.021)	0.808
Diastolic BP (mmHg)	0.984(0.956–1.012)	0.263	0.971(0.919–1.025)	0.287	0.994(0.949–1.042)	0.812
Dyslipidemia(Yes)	1.130(0.639–2.000)	0.674	0.623(0.218–1.779)	0.376	2.210(0.869–5.624)	0.096
Total cholesterol (mmol/L)	0.617(0.160–2.386)	0.484	1.227(0.095–15.873)	0.876	0.378(0.052–2.733)	0.335
Triglyceride (mmol/L)	0.731(0.489–1.093)	0.127	0.600(0.244–1.476)	0.266	0.805(0.489–1.327)	0.396
HDL-cholesterol (mmol/L)	0.288(0.057–1.466)	0.134	0.335(0.017–6.726)	0.474	0.588(0.051–6.842)	0.672
LDL-cholesterol (mmol/L)	2.066(0.392–10.888)	0.339	0.466(0.019–11.459)	0.640	3.843(0.322–45.803)	0.287
FPG (mmol/L)	1.300(1.135–1.488)	<0.001	1.499(0.775–2.900)	0.229	1.240(1.016–1.515)	0.035*
2hPG (mmol/L)	N/A*	N/A*	1.543(1.160–2.051)	0.003*	0.953(0.833–1.089)	0.477
HbA1c (%)	1.000(0.787–1.271)	0.999	0.613(0.200–1.879)	0.391	0.904(0.643–1.273)	0.564
Urinary ACR (Albuminuria)	1.496(0.979–2.286)	0.063	2.131(0.909–4.999)	0.082	0.847(0.452–1.585)	0.604
eGFR (MDRD equation)ml/min/1.73 m^2^	0.979(0.960–0.998)	0.029	0.997(0.962–1.034)	0.882	0.955(0.928–0.983)	0.002*
Duration of Diabetes(years)	N/A	N/A	N/A	N/A	1.098(1.052–1.146)	<0.001*

NGT, normal glucose tolerance; IGR, impaired glucose regulation; DM, diabetes mellitus; FPG, fasting plasma glucose; 2hPG,2-h postprandial plasma glucose; HbA1c, glycated hemoglobin A1c; HDL, high density lipoprotein; LDL, low-density lipoprotein; eGFR, estimated glomerular filtration rate; ACR, albumin-to-creatinine ratio;

The OR corresponds to a 1 unit change in categorical factors associated with PN;

N/A: not applicable.

* 2hPG levels were from OGTT (NGT, IGR and newly-diagnosed diabetes subjects), and 100g steamed bread meal test (Known diabetes subjects), so these data could not be combined in the regression analysis in total subjects.

The prevalence of PN increased with age (<50 years: 1.0%, 50–59 years: 1.3%, 60–69 years: 2.8%, and ≥70 years: 11.3%), diabetes duration (0 years: 2.5%, <5 years: 9.3%, 5–9 years: 11.9%, and ≥10 years: 18.9%), and increasing HbA1C levels (HbA1C levels <5.6%: 2.6%, HbA1C levels = 5.6%–6.5%: 3.2%, HbA1C levels = 6.5%–8.0%: 7.5%, and HbA1C levels ≥8.0%: 11.2%).

### Risk Factors for PN

The univariate regression models including the entire study population revealed significant differences between those with and without PN with respect to the following variables: age, odds ratio (OR) 1.100 (95% CI: 1.075–1.125); duration of diabetes, OR 1.134 (95% CI: 1.101–1.169); waist circumference, OR 1.045 (95% CI: 1.024–1.067); systolic blood pressure, OR 1.020 (95% CI: 1.010–1.030); hypertension, OR 2.159 (95% CI: 1.343–3.471); fasting plasma glucose, OR 1.192 (95% CI: 1.108–1.283); HbA1C levels, OR 1.422 (95% CI: 1.245–1.623); HDL-cholesterol, OR 0.455 (95% CI: 0.209–0.990); urinary ACR, OR 2.634 (95% CI: 1.846–3.757) and eGFR, OR 0.949 (95% CI: 0.934–0.965). There were no significant differences with respect to the female gender, current smoking status, diastolic blood pressure, dyslipidemia, and levels of total cholesterol, triglycerides, LDL-cholesterol, and HDL-cholesterol.

The final multivariate logistic regression models included age, the female gender, current smoking status, waist circumference, hypertension, systolic blood pressure, diastolic blood pressure, dyslipidemia, and levels of total cholesterol, triglyceride, HDL-cholesterol, LDL-cholesterol, fasting plasma glucose, 2-h postprandial plasma glucose, HbA1C, urinary ACR, eGFR, and duration of diabetes among the NGT, IGR, and diabetes subjects. The independent variables remaining in the final multiple logistic regression models with PN as the dependent variable are listed in Table 4. Among the IGR subjects, independent associations with PN were noted for age and 2-h postprandial plasma glucose levels (both *P*<0.05). Among the diabetes subjects, independent associations with PN were noted for fasting plasma glucose, duration of diabetes, and eGFR (all *P*<0.05); age was not significantly associated with PN (*P* = 0.110).

## Discussion

Many studies indicate that the prevalence of IGT is up to 40%–50% in idiopathic neuropathy patients [7], [8], [12]. This contrasts to a prevalence of approximately 15% in a similarly aged population [20]. An increased prevalence of IGR among subjects with idiopathic neuropathy suggests that PN is a continuous lesion and is present in some proportion of hyperglycemia patients.

The present results demonstrate that in the general Chinese population, the prevalence of PN is slightly higher in people with IGR than that in people with NGT. Similar to the study of Ziegler et al. [5], the present study does not corroborate the results of some previous studies indicating that IGR is associated with an increased prevalence of polyneuropathy [21], [22]. The marked variation in the prevalence of PN in IGR subjects might be due to many reasons. First, the present study indicates the prevalence of PN is relatively lower in NGT and IGT subjects; therefore, the relatively small sample size might be one of the reasons for the observed variation. Second, the disparate methods used to assess neuropathy might be another reason. Neural impairment in IGT subjects may be mainly subclinical, asymptomatic, and characterized by small-fiber neuropathy and mild impairment of cardiovascular autonomic function [12], [23]. If methods that predominantly detect large-fiber dysfunction are used, the subtle changes present in IGT may not be observed. In our study, definition of neuropathy is based on the modified NDS and the NSS, which is the lack of a rigorous assessment of small fibre damage. So this might explain the differences with other studies depending on the PN definition. Although methodological differences in the assessment of neuropathy make comparisons difficult, a previous study reports that NSS/NDS and NCV are concordant in the Chinese population [24]. In addition, the methods used in the present study were simple, convenient, and generally accepted in epidemiological studies. Finally, the study design, ethnic differences, and the age and sex structure of the study population itself might have contributed to the variation. For example, the prevalence of polyneuropathy in individuals with IGR and DM in the She ethnic minority group in China is higher than that in subjects with NGT [3].

The present results indicate that age and 2-h postprandial glucose levels are independently associated with PN in IGR subjects. It is conceivable that advanced age may contribute to the higher prevalence of PN in individuals with IGT than that in individuals with NGT, because age was associated with PN in the entire study population.

Furthermore, we showed for the first time that the 2-h postprandial glucose level is an independent risk factor for PN in Chinese subjects with IGR. The prevalence of polyneuropathy in individuals with IFG is marginally lower than that in those with IGT in the studies of Ziegler et al. [4], [5], considering that the 2-h postprandial glucose levels appear to be more closely associated with PN risk than the fasting glucose levels. Postprandial glucose levels may be involved in the pathogenesis of PN in IGR subjects [25], [26].

Neuropathy in IGT and diabetes seems to share similar pathogenetic mechanisms. Injury to small blood vessels leading to nerve ischemia, direct nerve toxicity due to episodic hyperglycemia and insulinopenia, increased oxidative stress, advanced glycation end-product accumulation, and impaired axonal transport all play a role in the pathogenesis of the neuropathy in IGT [27]. Hyperglycemia creates a proinflammatory microenvironment, and inflammatory processes and biomarkers of inflammation contribute to PN [28].In addition, deficits in neurotrophic factors such as nerve growth factor and changes in the secretion of neuroeffector peptides such as substance P and calcitonin gene-related peptide can be responsible for neuropathy.

The present study shows that subjects with PN had a higher waist circumference than those without PN among the subjects without diabetes. However, waist circumference was not an independent risk factor for PN in subjects with either IGR or diabetes according to the logistic regression analysis. Among the 1172 type 1 diabetes subjects without baseline neuropathy followed in the Eurodiab study, hypertension, smoking, obesity, and serum triglyceride levels were independent risk factors for neuropathy [29]. Furthermore, several small studies link obesity and idiopathic neuropathy. In another study, among 58 morbidly obese subjects, there was evidence of small fiber axonal dysfunction irrespective of the presence of diabetes and despite the fact that all but 4 subjects were asymptomatic [30]. Another small study of 20 obese subjects without diabetes and 20 age-matched control subjects demonstrates abnormal compound muscle and sensory nerve action potential amplitudes as well as abnormal sensory thresholds. However, many studies do not report any associations between BMI or weight and the prevalence of polyneuropathy in diabetes patients [31], [32]. In the Australian Diabetes Obesity and Life-style (AusDiab) study [33] on type 2 diabetes patients, neither BMI nor waist circumference was identified as a risk factor for polyneuropathy in the univariate analyses. Whether central obesity is a harbinger of polyneuropathy can only be determined by prospective studies involving subjects with diabetes and IGR.

The prevalence of DPN in the diabetes population in the present study was 8.4%, which is lower than that reported in any other population [29], [34]. The main reason for this is that 47% of the patients with diabetes in our study were newly diagnosed; the prevalence of DPN among them was only 2.9%. Diabetes patients, especially those with KDM, had the highest prevalence of DPN (13.1%) among all groups. Many previous studies on DPN report a wide range of prevalence estimates, from 7.8%–61.8% in different populations with type 2 DM [2], [29], [34]–[35]. The marked variation in the prevalence of neuropathy in the present study might be due to the diagnostic criteria of neuropathy, study design, sample selection, ethnicity, year when the study was conducted, and the age and sex structure of the study population itself.

The results of the present study corroborate the previously reported association between PN and glycemic control [2], [36]. As shown in the multivariate regression analysis (Table 4), the fasting plasma glucose level was an important independent risk factor for PN with an OR of 1.240 (95% CI, 1.016–1.515). UKPDS and DCCT confirm that long-term hyperglycemia can cause and accelerate chronic diabetes complications including PN and that intensive blood glucose control can decrease the incidence of PN by 40%. Early detection of PN in IGR and DM subjects is particularly important, and early glucose control will remarkably prevent or improve PN. The present results demonstrate that decreased eGFR is associated with PN, similar to the study of Kong et al. [37].

In contrast to some other studies, an important methodological strength of the present study is the use of the OGTT, similar with the KORA studies by Ziegler. This enabled us to study the entire spectrum of glucose disorders by identifying subjects with undiagnosed diabetes and pre-diabetes. Additionally, in the present study, many neurologic bedside tests, facilitating a relatively accurate definition of clinical PN, were used. Meanwhile, the major limitation of the present study is the criteria used to define neuropathy or in particular nerve damage. The NDS and NSS score is the lack of a rigorous assessment of small fibre damage which is thought to be the earliest nerve fibre deficit and the one likely to occur in IGR. The other limitation of the present study is that no inferences regarding cause and effect can be made because of the cross-sectional design.

### Conclusions

The present study was based on a large, community-based population of Han Chinese people and used well-accepted screening methods. The results show that the prevalence of PN in NGT, IGR, and DM subjects was 1.5%, 2.8%, and 8.4% respectively. The prevalence of PN is slightly higher in individuals with IGR than that in individuals with NGT, but small fibre damage in IGR as the earliest nerve fibre deficit may be underestimated in our study.The risk factors for PN in IGR subjects include age and postprandial glucose level, suggesting that the postprandial plasma glucose level is an important target for preventing or improving PN in IGR subjects.

## References

[1] Yang W, Lu J, Weng J, Jia W, Ji L, et al (2010) Prevalence of Diabetes among Men and Women in China. N Engl J Med 362: 1090–1101. 20335585 10.1056/NEJMoa0908292

[2] Lu B, Yang Z, Wang M, Yang Z, Gong W, et al (2010) High prevalence of diabetic neuropathy in population-based patients diagnosed with type 2 diabetes in the Shanghai downtown. Diabetes Res Clin Pract 88: 289–294. 20359765 10.1016/j.diabres.2010.02.002

[3] Lin Y, Xu Y, Chen G, Lai X, Huang B, et al (2012) Diabetes and its chronic complications in the she ethnic minority group of China. Diabetes Technol Ther 14: 430–439. 22304539 10.1089/dia.2011.0244 PMC3338954

[4] Ziegler D, Rathmann W, Dickhaus T, Meisinger C, Mielck A (2009) Neuropathic pain in diabetes, prediabetes and normal glucose tolerance: the MONICA/KORA Augsburg Surveys S2 and S3. Pain Med 10: 393–400. 19207236 10.1111/j.1526-4637.2008.00555.x

[5] Ziegler D, Rathmann W, Dickhaus T, Meisinger C, Mielck A (2008) Prevalence of polyneuropathy in pre-diabetes and diabetes is associated with abdominal obesity and macroangiopathy: the MONICA/KORA Augsburg Surveys S2 and S3. Diabetes Care 31: 464–469. 18039804 10.2337/dc07-1796

[6] Eriksson KF, Nilsson H, Lindgärde F, Osterlin S, Dahlin LB, et al (1994) Diabetes mellitus but not impaired glucose tolerance is associated with dysfunction in peripheral nerves. Diabet Med 11: 279–285. 8033527 10.1111/j.1464-5491.1994.tb00272.x

[7] Singleton JR, Smith AG, Bromberg MB (2001) Increased prevalence of impaired glucose tolerance in patients with painful sensory neuropathy. Diabetes Care 24: 1448–1453. 11473085 10.2337/diacare.24.8.1448

[8] Novella SP, Inzucchi SE, Goldstein JM (2001) The frequency of undiagnosed diabetes and impaired glucose tolerance in patients with idiopathic sensory neuropathy. Muscle Nerve 24: 1229–1231. 11494278 10.1002/mus.1137

[9] Sahin S, Karsidag S, Ayalp S, Sengul A, Us O (2009) Determination of nerve conduction abnormalities in patients with impaired glucose tolerance. Neurol Sci 30: 281–289. 19444381 10.1007/s10072-009-0089-8

[10] Isak B, Oflazoglu B, Tanridag T, Yitmen I, Us O (2008) Evaluation of peripheral and autonomic neuropathy among patients with newly diagnosed impaired glucose tolerance. Diabetes Metab Res Rev 24: 563–569. 18636432 10.1002/dmrr.859

[11] Green AQ, Krishnan S, Finucane FM, Rayman G (2010) Altered C-fiber function as an indicator of early peripheral neuropathy in individuals with impaired glucose tolerance. Diabetes Care 33: 174–176. 20040675 10.2337/dc09-0101 PMC2797968

[12] Sumner CJ, Sheth S, Griffin JW, Cornblath DR, Polydefkis M (2003) The spectrum of neuropathy in diabetes and impaired glucose tolerance. Neurology 60: 108–111. 12525727 10.1212/wnl.60.1.108

[13] Polydefkis M, Griffin JW, McArthur J (2003) New insights into diabetic polyneuropathy. JAMA 290: 1371–1376. 12966130 10.1001/jama.290.10.1371

[14] Guidelines Subcommittee (1999) 1999 World Health Organization-International Society of Hypertension Guidelines for the Management of Hypertension. J Hypertens 17: 151–183. 10067786

[15] Expert Panel on the Identification, Evaluation, and Treatment of Overweight in Adults (1998) Clinical guidelines on the identification, evaluation, and treatment of overweight and obesity in adults: Executive summary. Am J Clin Nutr 68: 899–917. 9771869 10.1093/ajcn/68.4.899

[16] American Diabetes Association (2012) Diagnosis and classification of diabetes mellitus. Diabetes Care 35: S64–S71. 22187472 10.2337/dc12-s064 PMC3632174

[17] Cabezas-Cerrato J (1998) The prevalence of clinical diabetic polyneuropathy in Spain: a study in primary care and hospital clinic groups. Neuropathy Spanish Study Group of the Spanish Diabetes Society (SDS). Diabetologia 41: 1263–1269. 9833931 10.1007/s001250051063

[18] Young MJ, Boulton AJ, MacLeod AF, Williams DR, Sonksen PH (1993) A multicentre study of the prevalence of diabetic peripheral neuropathy in the United Kingdom hospital clinic population. Diabetologia 36: 150–154. 8458529 10.1007/BF00400697

[19] Daousi C, MacFarlane IA, Woodward A, Nurmikko TJ, Bundred PE, et al (2004) Chronic painful peripheral neuropathy in an urban community: a controlled comparison of people with and without diabetes. Diabet Med 21: 976–982. 15317601 10.1111/j.1464-5491.2004.01271.x

[20] Harris MI, Hadden WC, Knowler WC, Bennett PH (1987) Prevalence of diabetes and impaired glucose tolerance and plasma glucose levels in the US population age 20–74 years. Diabetes 36: 523–534. 3817306 10.2337/diab.36.4.523

[21] Franklin GM, Kahn LB, Baxter J, Marshall JA, Hamman RF (1990) Sensory neuropathy in non-insulin-dependent diabetes mellitus: the San Luis Valley Diabetes Study. Am J Epidemiol 131: 633–643. 2316495 10.1093/oxfordjournals.aje.a115547

[22] de Neeling JN, Beks PJ, Bertelsmann FW, Heine RJ, Bouter LM (1996) Peripheral somatic nerve function in relation to glucose tolerance in an elderly Caucasian population: the Hoorn study. Diabet Med 13: 960–966. 8946154 10.1002/(SICI)1096-9136(199611)13:11<960::AID-DIA268>3.0.CO;2-Z

[23] Putz Z, Tabák AG, Tóth N, Istenes I, Németh N, et al (2009) Noninvasive evaluation of neural impairment in subjects with impaired glucose tolerance. Diabetes Care32: 181–183. 10.2337/dc08-1406 PMC2606810 18835942

[24] Jia WP, Shen Q, Bao YQ, Lu JX, Li M, et al (2006) Evaluation of the four simple methods in the diagnosis of diabetic peripheral neuropathy. Natl Med J China 86: 2707–2710. 17199983

[25] Bongaerts BW, Rathmann W, Kowall B, Herder C, Stöckl D, et al (2012) Postchallenge hyperglycemia is positively associated with diabetic polyneuropathy: the KORA F4 study. Diabetes Care 35: 1891–1893. 22751964 10.2337/dc11-2028 PMC3424984

[26] Papanas N, Ziegler D (2012) Polyneuropathy in Impaired Glucose Tolerance: Is Postprandial Hyperglycemia the Main Culprit? A Mini-Review. Gerontology. 2012 Dec 1. [Epub ahead of print]. 10.1159/000343988 23207896

[27] Smith AG, Singleton JR (2008) Impaired glucose tolerance and neuropathy. Neurologist 14: 23–29. 18195653 10.1097/NRL.0b013e31815a3956

[28] Nguyen DV, Shaw LC, Grant MB (2012) Inflammation in the pathogenesis of microvascular complications in diabetes. Front Endocrinol (Lausanne) 3: 170. 23267348 10.3389/fendo.2012.00170 PMC3527746

[29] Tesfaye S, Chaturvedi N, Eaton SE, Ward JD, Manes C, et al (2005) Vascular risk factors and diabetic neuropathy. N Engl J Med 352: 341–350. 15673800 10.1056/NEJMoa032782

[30] Herman RM, Brower JB, Stoddard DG, Casano AR, Targov-nik JH, et al (2007) Prevalence of somatic small fiber neuropathy in obesity. Int J Obes (Lond) 31: 226–235. 16770330 10.1038/sj.ijo.0803418

[31] Gregg EW, Sorlie P, Paulose-Ram R, Gu Q, Eberhardt MS, et al (2004) 1999–2000 national health and nutrition examination survey: Prevalence of lower-extremity disease in the U.S. adult population > = 40 years of age with and without diabetes: 1999–2000 National Health and Nutrition Examination Survey. Diabetes Care 27: 1591–1597. 15220233 10.2337/diacare.27.7.1591

[32] Cheng YJ, Gregg EW, Kahn HS, Williams DE, De Rekeneire N, et al (2006) Peripheral insensate neuropathy–a tall problem for US adults? Am J Epidemiol 164: 873–880. 16905646 10.1093/aje/kwj281

[33] Tapp RJ, Shaw JE, de Courten MP, Dun-stan DW, Welborn TA, et al (2003) Foot complications in type 2 diabetes: an Australian population-based study. Diabet Med 20: 105–113. 12581261 10.1046/j.1464-5491.2003.00881.x

[34] Davies M, Brophy S, Williams R, Taylor A (2006) The Prevalence, Severity, and Impact of Painful Diabetic Peripheral Neuropathy in Type 2 Diabetes. Diabetes Care 29: 1518–1522. 16801572 10.2337/dc05-2228

[35] Dyck PJ, Clark VM, Overland CJ, Davies JL, Pach JM, et al (2012) Impaired glycemia and diabetic polyneuropathy: the OC IG Survey. Diabetes Care 35: 584–591. 22355020 10.2337/dc11-1421 PMC3322692

[36] Liu F, Bao Y, Hu R, Zhang X, Li H, et al (2010) Screening and prevalence of peripheral neuropathy in type 2 diabetic outpatients a randomized multicentre survey in 12 city hospitals of China. Diabetes Metab Res Rev 26: 481–489. 20661939 10.1002/dmrr.1107

[37] Kong AP, So WY, Szeto CC, Chan NN, Luk A, et al (2006) Assessment of glomerular filtration rate in addition to albuminuria is important in managing type II diabetes. Kidney Int 69: 383–387. 16408130 10.1038/sj.ki.5000061

